# Progress of Veterinary Science

**Published:** 1881-01

**Authors:** 


### PROGRESS OF VETERINARY SCIENCE.

The Standard Ration for Horses, recommended by Dr. Wolff, of Hohenheim, as the result of a series of careful experiments, is as follows:

PER 1,000 LBS. LIVE WEIGHT.

| | Total dry matter. | Digestible. | | | Nutritive ratio. |
|---|---|---|---|---|---|
| | | Albuminoids. | Carbo-Hydrates. | Fat. | |
| | *lbs.* | *lbs.* | *lbs.* | *lbs.* | |
| Light work. . . . . . | 21.0 | 1.5 | 9.1 | 0.3 | 6.5 |
| Ordinary work . . . . | 22.5 | 1.8 | 11.2 | 0 6 | 7 0 |
| Heavy work. . . . . | 25 5 | 2.8 | 13.4 | 0.8 | 5.5 |

Spurious Pregnancy in the Lower Animals.—The Rev. Dr. Haughton relates a singular case of spurious pregnancy in an ass. The animal was placed with a zebra, by which it was, during heat, frequently covered. After this connection, the abdomen of the animal began to enlarge, the mammary glands to harden and increase in size. There was no further return of heat. The animal was believed by all to be pregnant. At the end of eleven months, however, which is the regular duration of its pregnancy, the enlargement quite rapidly disappeared, and the animal returned to its normal size and condition. The ass was put with the zebra again, and the same result followed.

Dr. Haughton thought that there might be some psychological element in producing this spurious pregnancy. The lower animals had not, he thought, been credited with the intelligence which was their due, and sexual passion and instinct were in many very strong.

The Gastrodiscus Sonsinoii (Cobbold)—A New Parasite of the Horse.—Equine parasites belonging to the order of trematoda are quite rare. The gastrodiscus is homologous to the *amphistomum conicum*, found in the digestive organs of cattle and much less dangerous than the former. The gastrodiscus was discovered about 1876, in a horse, in Egypt, by an Italian veterinarian practising at Cairo. This gentleman, a Dr. Sonsino, sent specimens of the worm to Cobbold, of London, and Leuckart, of Leipsig. By these helminthologists the worm was found to belong to an entirely new species, of the order trematoda and the group of amphistomum. The worm received the name of *gastrodiscus* (Leuckart), or *gastrodiscus sonsinoii* (Cobbold). The parasite had a rounded, disc-like form. Its ventral surface was concave, and was covered by hundreds of little sucking papillæ. The dorsal surface was smooth and bulging. Anteriorly a buccal sucking apparatus was found at the end of a small cylindrical neck. At the opposite end a second, much larger, sucking apparatus was seen. The latter occupied the dorsal aspect.

This parasite has again been found in the body of a mule, that died so rapidly as to suggest the probability of poisoning. Two other animals had died before it. Countless worms, representing the parasite under consideration, were found to line the mucous membrane of the alimentary canal, from the pharynx to the anus. M. Poirier and M. Mégnin examined the worms carefully, and were thus able to somewhat modify and correct the description given by Cobbold.—*Gaz. Méd. de Paris*, quoted in *Annales de Méd. Vétér.*, October, 1880.

Experiments in Horse Feeding.—A singular experiment is at present under trial in the French army. The Minister of War, anxious to reduce the bulk of provisions to be carried during a campaign, has ordered that a new kind of biscuit should be used as food for the horses. On the conclusion of the present manœuvres this food will be rigidly tested. Two squads of cavalry and a battery of artillery will at once be put under the new system. The circular of the Minister directs all the details, and orders that a commission composed of four officers and a veterinary surgeon should see that the instructions are completely carried out, and report on their progress and results. The selected horses are to be weighed and measured across the shoulders before the experiment commences, and two other squadrons of cavalry and a battery of artillery are to be selected, composed as nearly as can be of animals of the same weight and size. They are to be placed under allowance of fodder, and both at once to be subjected to the estimate averages of a campaign during twelve days. The experiment will be followed out, while the officers charged with its conduct will constantly report on its result. The horses are to be supplied with new food on the first day of the campaign, without any transition or preparation for the change. The biscuits are to be watered before each meal.

Cows Remaining in Heat.—Sometimes it happens that cows continue in heat for weeks, or even months, although, meanwhile, they may have taken the bull a number of times. In rare cases, the heat continues even though impregnation has taken place; but generally the cow will not breed. Animals in this condition are a great annoyance. The best plan is to give them a cathartic after a day's fast; then turn them out with a bull, and keep them on a low diet, with large doses of bromide of potassium.

Malignant Pustule and the Œdema of Anthrax.—M. Colin, of Alfort (*Annales de Médecine Vétér.*, Sept., 1880), has recently undertaken an experimental analysis of the various forms and degrees of virulence of anthrax. He found that certain animals would not, after inoculation with the virus taken from the human being, develop the malignant pustule as observed in man. On the other hand, certain other animals showed different degrees of infection, and exhibited the disease in various distinctly characterized forms. If the blood of an infected being was inoculated into the skin of animals not apt to contract anthrax, the following different results were at various times obtained:

1. A simple half-closed opening, which, for several days, enclosed the virulent substance.
2. Diffuse erythema of short duration, disappearing quite suddenly.
3. Simple malignant pustule, projecting more or less. It was always red, umbilicated, and at first showed a serous discharge, which later became sero-purulent.
4. Simple œdema, without further cutaneous change.
5. Pustule followed by œdema.
6. Œdema followed by a pustule.
7. Pustule and œdema simultaneously developed.
8. Pustule with erythema and œdema.
9. A phlegmonous tumor.
10. Simple bubo, with lymphangitis of adjoining tissue.

The dog dees not readily acquire these various manifestations of successful inoculation, and here they usually have a benign character; whereas, in the human being, the affection is commonly of the malignant type. Never-the less, the dog's pustule shows its virulent properties when some of its secretion is tried on other animals. The mortality of the dogs was greatest in young subjects. Altogether, 23 dogs died out of 153 experimented upon. Of course, no treatment was resorted to. The mortality, therefore, only reached 14 per cent. In 91 animals, no appreciable effects resulted from the inoculation.

The Latest Theory Regarding the Cause of Abortion in Cows.—The latest theory in regard to abortion in cows is that put forth by Mr. Clark, of Battle Creek, Mich., in his work, recently published, entitled "Cattle Problems Made Clear." He does not consider abortion a disease in the ordinary sense of the term, but the "result of serious structural derangement in the size and the use of the mammary arteries—those that lead to the milk glands—the derangement resulting from the engorgement and relaxation of those arteries which, on account of their extremely enlarged size, convey so much of the mammary blood to the udder, that not enough is left to increase the necessary supply to the placenta, or continue the growth of the embryo; hence its death from deficiency of nourishment, and its expulsion a necessity to save the cow." He argues that milking pregnant cows too late, is antagonistic to their breeding power, and that a large yield of milk during pregnancy tends to dwarf embryo growth. Cows whose large capacity for yielding milk has been developed *gradually*, through many generations it would seem, on the hypothesis of Mr. Clark, would not be as liable to abort as common or ordinary cows that had been put through a forcing system; for in the latter case the increase of milk has been developed too rapidly. Hence, in the language of Mr. Clark, "abortion results from engorgement of the arteries that supply the udder glands with blood, the too great and rapid increase of blood being the result of equally great and rapid increase in the *feed* of the cows, whose arteries are consequently engorged, and their udders similarly filled and engorged with milk formed from a sudden increase in the quantity of arterial blood supplied to the milk glands." Mr. Clark treats the subject at considerable length in his book, and suggests means for the prevention of abortion upon this view of its cause.

RICKETTY EGGS.—It occasionally happens that hens will persist in laying thin-shelled eggs. The trouble has naturally been ascribed to a deficiency in lime salts, and the fowls are supplied with oyster shells, crushed bones, lime, ashes, etc. But these things very often do no good whatever, while, on the other hand, a careful attention to food, air, exercise, and sunlight will bring about a cure. This fact will be received with much satisfaction by those who do not believe in the prevalent theories regarding the pathology and therapeutics of rickets.

SCURFY LEGS IN FOWLS.—During the moulting season the legs undergo the same process of change, and a new skin grows, which, if the legs be without scruf, will appear bright and fresh like a chick's. There is nothing more unsightly than scurfy legs. This is in part hereditary, and in part brought on by filth. None but white and yellow-legged birds are affected with it to any extent. Although scurfy legs may be cured by the use of carbolic soap and an ointment prepared of lard and sulphur, it is better, if possible, to avoid the trouble by breeding and careful cleanliness. To shun bumble-footedness and bunchy toes, or lumps on the web, train the birds to low roosting poles, and keep their walks comparatively clean.—*Country Gentleman.*

BUMBLE-FOOT.—Most of the ailments that appear in the feet of fowls may be traced to some local cause. Bumble-foot and scurfy legs are not uncommon. The health and profit of the fowls thus afflicted may not be injured by these unsightly diseases, but it is better to preserve clean shanks and perfect feet. Bumble-foot is caused by a large bunch gathering on the sole or heel of the foot, which may or may not suppurate and disappear in the course of time. Oftener it turns into a hard callus, which contains no pus, and should be left alone. The origin of this difficulty is a bruise, caused by alighting from high perches, or sudden landing from high flights. It is seen mostly in large or heavy birds. It is rare in the small breeds. The best way is to prevent the trouble by proper arrangement of the roosting poles. Round perches are preferable to flat or square strips. The poles should be small—for a full-grown fowl not more than an inch and a half in diameter. A large fowl will roost on a small branch. One that is sufficiently large to bear the weight is all that is necessary. When roosting on a tree, fowls generally seek the outer branches, that are small and easy to clasp the toes around. Square or large perches cause crooked breast-bones, produced when young while the bone is yet gristly.

Where many fowls roost in a building, there cannot be allowed much vacant space. Where this is the case, have the poles small, but place frequent supports underneath to bear the weight, and be sure they are firm and reliable. Bumble-foot is often contracted from perching on a flat pole, where the toes must be extended, and the whole weight comes on the ball of the foot. The position is unnatural and uncomfortable. A little care in this respect will avoid many unsightly bunches on the feet.—*Country Gentleman.*

Fowl Cholera in the West.—Dr. Horne, in a letter to the *Cultivator and Country Gentleman*, says, regarding fowl cholera:

"I have received many letters of inquiry respecting the treatment of this formidable disease. Many persons write, saying that they have lost all their fowls, of mixed kinds, and with various roosting places. Those well housed suffered about the same as those which improvised a roosting place on trees, sheds, fences, etc. Carefully studying all the conditions as I have received them lately, there would appear to be none which seemed to be of any benefit to poultry. Some of the best regulated yards seem to have suffered quite as much as any. I have visited some of the best, and found the ravages of the disease to be as bad as I found anywhere. Not only do methods of housing make little or no difference, but I find that food also seems of little influence. I found the death rate as great where mixed food was systematically given as I did where only one general food was given. I found no more losses where moderately confined than I did where the fowls had an unlimited range of a whole farm.

"I found that all sorts of medicines had been given, according to the belief or fancy of the person in charge. All seemed to be useless, and the disease is on the increase here in the Northwest. Well, what are we to do?

I hardly know myself; but as my yards are so far unaffected, while the disease is all around me, there must be something in my management. I give plenty of air, keep clean, and feed half a dozen kinds of food. I keep all the places where the droppings can fall constantly disinfected with kerosene. I sprinkle all the places each time I clean them out (twice a week). As soon as I see the slightest drooping or discoloring of the comb, I wash in kerosene and give them five drops of tincture of nux vomica in half a teaspoonful of water. All the water they drink should be impregnated with iron. I give mine in an old rusty ash drawer from an old coal stove, which answers well."

Dyspepsia in Pigs.—When pigs are affected with indigestion, salt and charcoal will be found a remedy. Mix the salt and charcoal together, putting them in a box accessible to the pigs; they will eat no more than required.

Milk Sickness, or Trembles.—A cow affected with milk sickness will communicate the disease, through her milk, to her calf; the calf dying, will give the disease to hogs eating the dead calf; dogs eating dead hogs will die of the disease. It is a disease that reproduces the cause in the animals affected. This would be rather against the theory of mineral poison. Impure drinking water may cause it.—*Cor. Phila. Med. and Surg. Reporter.*

The Embryo of the Tapeworm.—M. Poincaré, of Nancy, has recently announced (*Comptes Rendus*) that he has discovered a new parasite, which he describes as an embryo of tænia. This parasite has heretofore been known simply as the corpuscle or utricle of Moscher. The supposition that it is the larva of tænia is novel, but it helps to explain the noxious power of beef in which no tænia are found. The most persevering search rarely discovers the unarmed cysticerci even in the beef of countries where tænia (*t. mediocanellata*) extensively prevails.

The Effect of Alcohol on Pigs.—At the meeting of the Medical Section of the French Association for the Advancement of Science, MM. Dujardin-Beaumitz and Audigé reported the results of their experiments with alcohol on pigs. They wished to see what would be the effect of the continuous use of small doses of the drug, and gave daily in the food from 1 to 2.7 grams (gr. xv. to gr. xl.) per kilogram (2 lbs.) weight of the animal. This amounted to above 200 grams per day. After keeping this up for a year, they found it had produced no visible change. But they did not think that enough time had elapsed to form any definite conclusions. In the hog, large doses of alcohol do not produce excitement, but simply causes a deep and quiet sleep.

